# Factors associated with use of community mental health services by schizophrenia patients using multilevel analysis

**DOI:** 10.1186/1472-6963-11-257

**Published:** 2011-10-07

**Authors:** Berta Moreno-Küstner, Fermín Mayoral, Fabio Rivas, Pedro Angona, Javier Requena, José M García-Herrera, Desiree Navas, Patricia Moreno, Antoni Serrano-Blanco, Juan A Bellón

**Affiliations:** 1 Research Unit Distrito Sanitario Malaga, IMABIS Fundation. Department of Personality, Evaluation and Psychological Treatment, University of Malaga, Spain. (Research network "redIAPP", "SAMSERAP" group; 2 Psychiatric Service, University Hospital Carlos Haya, Malaga, Spain, (Research network "redIAPP", "SAMSERAP" group; 3 Psychiatric Service, University Hospital Carlos Haya, Malaga, Spain; 4 Community Mental Health Centre "Guadalmedina". University Hospital Carlos Haya, Malaga, Spain (Research network "redIAPP", "SAMSERAP" group; 5 Community Mental Health Centre "Centro". University Hospital Carlos Haya, Malaga, Spain, (Research network "redIAPP", "SAMSERAP" group; 6 Research Unit Distrito Sanitario Malaga, IMABIS Foundation, Malaga, Spain, (Research network "redIAPP", "SAMSERAP" group; 7 Research and Development Unit, Parc Sanitari Sant Joan de Déu, Sant Boi de Llobregat, Barcelona, Spain, (Research network "redIAPP", "SM-SJ de Déu" group; 8 Health Centre El Palo. Research Unit Distrito Sanitario Málaga (Research network "redIAPP", "SAMSERAP" group). Department of Preventive Medicine, University of Malaga, Spain

## Abstract

**Background:**

Persons with schizophrenia and related disorders may be particularly sensitive to a number of determinants of service use, including those related with illness, socio-demographic characteristics and organizational factors. The objective of this study is to identify factors associated with outpatient contacts at community mental health services of patients with schizophrenia or related disorders.

**Methods:**

This cross-sectional study analyzed 1097 patients. The main outcome measure was the total number of outpatient consultations during one year. Independent variables were related to socio-demographic, clinical and use of service factors. Data were collected from clinical records.

**Results:**

The multilevel linear regression model explained 46.35% of the variance. Patients with significantly more contacts with ambulatory services were not working and were receiving welfare benefits (p = 0.02), had no formal education (p = 0.02), had a global level of severity of two or three (four being the most severe) (p < 0.001), with one or more inpatient admissions (p < 0.001), and in contact with both types of professional (nurses and psychiatrists) (p < 0.001). The patients with the fewest ambulatory contacts were those with diagnoses of persistent delusional disorders (p = 0.04) and those who were attended by four of the 13 psychiatrists (p < 0.001).

**Conclusions:**

As expected, the variables that explained the use of community service could be viewed as proxies for severity of illness. The most surprising finding, however, was that a group of four psychiatrists was also independently associated with use of ambulatory services by patients with schizophrenia or related disorders. More research is needed to carefully examine how professional support networks interact to affect use of mental health.

## Background

After deinstitutionalization, research on psychiatric services has focused on community care [1-6]. In most developed countries the majority of people with severe mental illness, mainly schizophrenia, are treated by community mental health services, with hospital admission when necessary [7]. Persons with schizophrenia and related disorders may be particularly sensitive to a number of determinants of service use [7]. Besides illness-related factors (diagnosis, severity and level of function), several studies highlight the individual and social determinants of mental health care use, including age, educational attainment [8,9], gender [10], ethnic affiliation [11], language used in ethnic minorities [12], socio-economic level [13,14], marital status, employment situation and urban or rural residence [15-17]. Other factors, such as the professionals attending the patients, can also be considered [18]. Thus, the organization of the mental health care services is instrumental in patterns of use by people with schizophrenia.

As Becker and Kilian [19] state, it is necessary to widen the scope of mental health service research towards a multilevel perspective including patient characteristics, health care services organization, professional profile and economic and socio-cultural environment [20], particularly in community-based mental health services [21].

We undertook a clinical and epidemiological study to identify factors associated with the number of contacts with the ambulatory mental health care team by patients with schizophrenia or related disorders.

## Methods

### Design and setting

This cross-sectional study was carried out in the Mental Health Department of Carlos Haya hospital. The hospital covers a geographically defined area in the province of Malaga (southern Spain), with a population in 2006 of 346,504 inhabitants. The mental health care department itself comprises two community mental health centres, one day centre, one general-hospital psychiatric unit (with 45 beds), one medium and long-stay ward, and one child and adolescent mental health unit. Multidisciplinary teams work in both community and hospital settings. The staff of the two community mental health centres is composed of 13 psychiatrists, 6 psychologists, 12 nurses (including auxiliary nurses), 2 social workers and 6 administrative staff. Patients attending the community mental health services are referred by their general practitioner or by emergency services or after discharge from an acute ward at the general hospital. The Spanish National Health Service provides free medical cover to 100% of the population. The study area includes 13 primary health care centres.

Cases were selected from "The Malaga Schizophrenia case-register: RESMA" which in 2003 started to identify all patients with schizophrenia or related disorders attended in this area. The RESMA collects cases from two large clinical databases of mental health services provision, one from an outpatient setting: SISMA (Mental Health Information System) and the other from an inpatient setting: CMBD (Minimum Basic Data Set). More details are explained elsewhere [22].

This study included patients with a clinical diagnosis of schizophrenia or related disorders (ICD-10 codes F20 to F29) in contact with the Community Mental Health Centres (Guadalmedina and Centro) of the Mental Health Department of Carlos Haya hospital, during 2006.

### Measures

The outcome measure was the number of outpatient contacts with community mental health services over a year. Outpatient contacts were defined as daily contacts (one or more) with staff at the community mental health care facility. As patients who were in hospital could not have any outpatient contact during the period of hospitalization, we calculated our dependent variable by dividing the total number of contacts over 1 year by the number of days the patient was not in hospital. For example, for a patient who had 20 contacts with the community centre during the study year and no inpatient episode, the figure was calculated by dividing the 20 contacts into 365 days. However, for another patient with the same number of contacts, 20, but who had spent 15 days in a hospital psychiatric ward, the figure was calculated by dividing the 20 contacts into just 350 days (365-15). Thus, the number of contacts with the ambulatory services was calculated in relation to the number of days a patient was at risk for such contacts.

### Independent variables

The following socio-demographic variables were included: age, gender, marital status (single, married/living with partner, separated/divorced, widowed), level of education [no schooling, primary (to 14 years of age), secondary (from 14-18 years), higher (bachelor's degree and higher)], type of living arrangement (alone, original family/other relatives and friends, own family, sheltered accommodation and homeless) and employment status (employed, unemployed, student, looking after family or house, not working receiving welfare benefits and other). Concerning place of residence, two variables were included: residence catchment area according to the area of the community mental health centre (Gualdamedina, Centro, or outside the study area), and municipality of residence, classified according to the rurality index developed for the Spanish population [23,24], which ranges from a minimum rurality factor of -3.59 to a maximum of 3.78 (Malaga city: -1.79, Rincon de la Victoria: -1.78, other villages: -1.59 to -0.61, ranked from less to more rurality).

Two clinical measures were available for this study population: the main clinical diagnosis and the Global Level of Severity. We grouped the clinical diagnoses of schizophrenia and related disorders corresponding to the codes F20-F29 of the International Classification of Diseases 10^th ^revision (ICD-10) [25] into five groups (F20: schizophrenia, F22: persistent delusion disorders, F23: acute and transient psychotic disorders, F25: schizoaffective disorders, and the last grouping of F21, F24, F28 and F29: schizotypal disorder, induced delusional disorder, other non-organic psychotic disorders and unspecified non-organic psychosis). The Global Level of Severity (GLS) is an index assigned to patients with schizophrenia or related disorders by psychiatrists, who usually treat patients according to symptom severity, disease evolution, social adjustment, disability level and treatment adherence. This index is used by psychiatrists in the clinical setting of the public mental health services in order to include patients in the category of "Severe Mental Illness" and thus assign more resources [22]. The GLS ranges from less severe (level I) to more severe (level IV) (Table 1). We calculated the test-retest agreement of the GLS using the intraclass correlation coefficient (ICC) because this index includes more than two levels [32]. The sample size was calculated to be sufficient for the intraclass correlation coefficient to be between plus or minus 0.10, which is considered a reasonable interval. Thus, the minimum number of patients to interview was 96 (9%), considering the ideal number to be 257 (24%). BM undertook the patient selection by simple random sampling from the list of patients under each professional. Later, each professional was given the list of his or her patients who had been selected to do the test-retest. Finally, we assessed a sample of 164 patients. The mean number of days between test and retest was 21 (range between 15-30 days). The ICC was 0.79 (95% CI, 0.71-0.84). This good coefficient means that the index was stable over the three week period.

**Table 1:** Description of Global Severity Index by levels

Levels	Description of each level
I	Clinically stable patient, without serious residual symptoms, with good social and family adjustment, autonomous or with minimal dependency, in regular contact with services
II	Patient with some symptoms, with evident disability or unstable evolution, who decompensates frequently, with moderate disability, adequate social support and contact with health services
III	Patient with predominant negative symptoms or residual symptoms with severe impairment of functioning, or with low social support or irregular service contact (non adherence to treatment)
IV	Patient with serious symptoms that jeopardize the patient's health or other people, with great disability, without social support and with poor contact with services or who refuses treatment

For use of services we included types of professional in the mental health centre during the study year (psychiatrist only, nurse only, both psychiatrist and nurse, and other professionals such as social welfare officer or psychologist). We included psychologist in the category of other professionals because in our mental health care organization psychologists do not normally look after schizophrenic patients. We also included in the analyses each psychiatrist (n = 13) working in the two community mental health teams who was responsible for the patients. In addition, inpatient status was defined as admittance to the psychiatric ward at the general hospital. Three different measures were used during 2006: a) number of admissions (0, 1, and 2 or more admissions); b) number of readmissions, defined for discharged patients if they were readmitted within 30 days after discharge (0 readmissions; 1 or more readmissions) and c) cumulative duration of all admissions during the study year (0 days, less than 19 days or more than 19 days). We chose this cut-off because it is the average length of stay in the psychiatric ward at the general hospital for schizophrenia patients in Andalusia [26].

### Statistical analysis

Missing data for independent variables were imputed using the method of multiple imputation through chained equations (MICE) implemented in the STATA "mi" program [27]. We imputed 20 data sets [28] and obtained combined estimates [29]. The number of missing data for independent variables is shown in Table 2.

**Table 2:** Demographic and clinical characteristics of outpatients with schizophrenia and related disorders attended in the mental health area of Carlos Haya Hospital

DEMOGRAPHIC CHARACTERISTICS	Patients N (%)	Outpatient contacts Mean SD
Age (mean ± SD years) 43.9 ±13.23		
**Sex**		
Male	691 (63.0)	11.71 ± 13.06
Female	406 (37.0)	11.00 ± 14.96
**Marital status**		
Single	626 (62.7)	13.18 ± 15.11
Married/with partner	230 (23.0)	7.30 ± 7.66
Separated/divorced/widowed	143 (14.3)	12.27 ± 5.36
(Missing data: 98)		
**Level of education**		
Primary School	456 (46.9)	12.13 ± 14.69
Secondary School	249 (25.6)	12.76 ± 15.24
No formal education and illiterate	166 (17.1)	9.59 ± 10.42
Higher education (Bachelor's degree)	102 (10.4)	10.75 ± 13.07
(Missing data:124)		
**Type of living arrangement**		
Original family/other relatives or friends	557 (56.1)	12.73 ± 15.49
Own family	267 (26.8)	7.79 ± 8.94
Alone	88 (8.8)	14.32 ± 13.71
Sheltered accommodation	72 (7.2)	14.64 ± 16.04
Homeless	11 (1.1)	13.19 ± 11.22
(Missing data:102)		
**Employment status**		
Not working, receiving welfare benefits	416 (43.4)	14.16 ± 16.69
Employed	174 (18.1)	8.54 ± 8.95
Unemployed	146 (15.2)	12.01 ± 12.80
Other	95 (9.9)	10.67 ± 14.16
Looking after family or home	76 (7.9)	7.29 ± 7.94
Student	53 (5.5)	11.47 ± 12.20
(Missing data: 137)		
**Residence catchment area**		
Centro		12.19 ± 13.42
Guadalmedina		11.77 ± 14.90
Outside study area		7.5 ± 8.21
**Municipality of residence **(ordered by rurality factor*)		
Malaga (city) (-1.78: less rurality)	841 (76.7)	12.39 ± 14.7
Rincon de la Victoria (-1.78)	89 (8.1)	10.12 ± 11.66
Other villages (-1.59 to -0.61: more rurality)	42 (3.8)	7.13 ± 8.6
Outside study area	125(11.4)	7.53 ± 8.21
CLINICAL CHARACTERISTICS		
**ICD-10 Clinical diagnosis**		
F20 Schizophrenia	676 (61.6)	12.73 ± 14.58
F22 Persistent delusional disorders	188 (17.1)	6.41 ± 6.95
F23 Acute and transient psychotic disorders	103 (9.4)	9.87 ± 10.06
F25 Schizoaffective disorders	81 (7.4)	15.73 ± 18.07
F21, F24, F28, F29 Schizotypal disorder, Induced delusional disorder, other non-organic psychotic disorders and unspecified non-organic psychosis	49 (4.5)	9.34 ± 15.49
**Global Level of Severity**		
Level I (less severity)	471 (50.6)	8.52 ± 9.32
Level II	344 (36.9)	13.47 ± 16.59
Level III	101 (10.8)	20.64 ± 17.05
Level IV (more severity)	16 (1.7)	16.38 ± 12.28
(Missing data:165)		

Distribution of patients (N = 1097) and outpatient contacts with community health services over one year

* -3.59 minimum to 3.78 maximum rurality factor

We performed multilevel linear regressions to test the hierarchical data structure, with the logarithm of the number of outpatient contacts to community mental health services over 1 year as the dependent variable. Multilevel logistic regression analyses were tested as the data structure was hierarchical, that is, patients could be grouped by psychiatrist and psychiatrists by health centre, though all were similar participants in the same group. The likelihood-ratio test of the null model with primary care centre as a random factor versus usual linear regression was significant (χ^2 ^= 24.19, P < 0.0001) and its variability was considerably higher than when using the psychiatrist as random factor (χ^2 ^= 13.79, P < 0.0001). However, the psychiatrist was used as a fixed effect variable in the model. Hence, we used multilevel linear regression with two levels, patients and primary care centre. With this model we performed bivariate analyses. We included all independent variables measured in the full model, and then excluded (step by step) from the model those variables that obtained a significance at P > 0.30. As the findings from these analyses were broadly similar, results from the full model are presented here. However, we removed from the model those variables that presented 0. The usefulness of including first-degree interactions in the equation was also considered. We repeated the analyses in participants with complete data as a sensitivity analysis. We conducted all analyses using STATA, release 11 [27].

## Results

### Sample description

In 2006, the RESMA included 1252 patients with diagnoses of schizophrenia or related disorders (codes F20-F29 of ICD-10). Of these, 118 never attended the ambulatory services at all as they were either seen only at the day hospital or in the acute hospital ward, and the number of contacts was unknown for another 37 patients. These two groups of patients were excluded from the analysis, giving a final data set of 1097 participants. Table 2 shows the demographic and clinical characteristics of the sample. In brief, cases were more frequently men, middle aged (average: 43.9 years old; SD: 13.23), single, with just primary education, living with their parents or other relatives and not working receiving welfare benefits. Most of the patients were living in Malaga city. The main diagnosis was schizophrenia (F20) and the patients mainly had the lowest Global Level of Severity, corresponding to level I. Table 3 shows the use of services by the patients. Concerning the type of professional who attended the patients, nearly half the patients were seen by both types of professional (psychiatrists and nurses). The distribution of the patients among the 13 psychiatrists was not homogenous, ranging from one psychiatrist who saw 13.6% of the patients to one who saw the fewest, only 2.6%. Finally, concerning inpatient psychiatric most of the patients had no hospital admissions. Only 14 patients had one or more readmission. Concerning the length of hospital stay, the average stay was less than 19 days for 46 patients. Finally, for the outcome measure, the average number of outpatient contacts over the one-year period was 11.44 (SD = 13.79).

**Table 3:** Use of services by outpatients with schizophrenia or related disorders attended in the mental health area of Carlos Haya Hospital

	Patients N (%)	Outpatient contacts Mean ± SD
OUTPATIENT CONTACTS		
**Type of professional who attended patient**		
Only psychiatrist	492 (44.8)	3.91 ± 2.50
Both psychiatrist and nurse	519 (47.3)	19.11 ± 16.37
Only nurse	78 (7.1)	8.95 ± 9.50
Other professionals (not psychiatrist or nurse)	8 (0.7)	2.01 ± 1.08
**Psychiatrist who attended patients**		
A	150 (13.7)	7.90 ± 7.49
B	131 (11.9)	11.70 ± 12.78
C	114 (10.4)	13.44 ± 14.82
D	109 (9.9)	13.64 ± 19.31
E	105 (9.6)	10.68 ± 16.12
F	96 (8.8)	12.99 ± 18.17
G	95 (8.7)	10.50 ± 10.70
H	93 (8.5)	14.71 ± 12.12
I	55 (5.0)	9.80 ±12.26
J	48 (4.4)	9.33 ± 9.78
K	39 (3.6)	9.77 ± 10.70
L	34 (3.1)	9.96 ± 9.68
M	28 (2.6)	13.56 ± 14.93
INPATIENT PSYCHIATRIC ADMISSIONS		
**Number of admissions**		
0 admission	1027 (93.6)	10.45 ± 12.71
1 admission	43 (3.9)	22.69 ± 15.67
2+ admissions	27 (2.5)	31.54 ± 23.92
**Number of readmissions**		
0 inpatient admission, 0 readmission	1027 (93.6)	10.45 ± 12.71
Any inpatient admission, 0 readmission	56 (5.1)	24.61 ± 18.63
Any inpatient admission, 1+ readmissions	14 (1.3)	32.09 ± 22.80
**Number of days in hospital** (Mean ± SD; 28.36 ± 25.54)		
0 day	1027 (93.6)	10.45 ± 12.71
< 19 days	46 (4.2)	24.58 ± 17.75
19+ days	24 (2.2)	29.03 ± 22.83

Distribution of patients (N = 1097) and outpatient contacts with community health services over one year

### Variables associated with number of ambulatory contacts

Table 4 shows the unadjusted analysis and the final adjusted analysis. The full multilevel linear regression model explained 46.35% of the estimated variance. Eight variables (sex, age, marital status, readmission episodes, number of days in hospital, type of living arrangement, residence catchment area and residence municipality) were no longer significant in the adjusted model. Five variables showed a significant association with a higher "number of ambulatory contacts during one year": a) type of professional: patients seen by both types of professional (nurses and psychiatrists) compared to those who only saw a psychiatrist; b) global level of severity: levels three and two compared with level one (less severity); c) number of psychiatric inpatient admissions: patients with one or more than one admission compared to those with no inpatient admissions; d) occupation status: patients who were not working and receiving welfare benefits compared to those who were employed; e) level of education: patients with no formal education or who were illiterate as compared to those with primary school level. Two variables were associated with a lower number of ambulatory contacts over the year: f) the particular psychiatrist: those patients seen by four specific psychiatrists (codes H, F, I, A) out of the 13 had fewer contacts as compared with the reference psychiatrists and; g) the diagnosis: patients with persistent delusion disorders compared with schizophrenia.

**Table 4:** Multilevel linear regression of outpatient contacts of schizophrenia patients, N = 1097

	Unadjusted model estimate				Adjusted model estimate			
	Coef.	SE	t	P value	Coef.	SE	t	P value
**Sex** (male)	0.17	0.06	2.69	0.00**	0.00	0.05	-0.04	0.97
**Age** (years)	-0.52	0.10	-5.10	0.00**	-0.12	0.11	-1.15	0.25
**Marital status**								
Single #	1.00				1.00			
Married/with partner	-0.45	0.08	-5.80	0.00**	-0.04	0.09	-0.42	0.67
Separated/divorced/widowed	-0.20	0.09	-2.09	0.04**	0.01	0.08	0.07	0.95
**Employment status**								
Employed #	1.00				1.00			
Unemployed	0.27	0.11	2.27	0.02**	0.02	0.09	0.30	0.76
Student	0.13	0.15	0.89	0.38	0.02	0.12	0.16	0.87
Looking after family or home	-0.18	0.14	-1.27	0.20	0.15	0.12	1.30	0.19
Not working, receiving welfare benefits	0.34	0.09	3.64	0.00**	0.19	0.08	2.34	0.02**
Other	0.05	0.13	0.41	0.68	-0.03	0.10	-0.30	0.76
**Level of education**								
Primary School #	1.00				1.00			
Secondary School	0.27	0.08	3.42	0.00**	0.11	0.06	1.68	0.09
No formal education and illiterate	0.32	0.09	3.57	0.00**	0.17	0.46	2.29	0.02**
Higher education (Bachelor's degree)	0.10	0.12	0.86	0.39	0.10	0.98	1.07	0.28
**Type of living arrangement**								
Original family/other relatives or friends #	1.00				1.00			
Own family	-0.38	0.08	-4.95	0.00**	-0.01	0.09	-0.18	0.86
Alone	0.06	0.12	0.52	0.60	0.18	0.09	1.96	0.05
Sheltered accommodation	0.04	0.13	0.27	0.78	0.00	0.10	0.05	0.96
Homeless	-0.15	0.24	-0.62	0.54	0.18	0.15	1.17	0.24
**Residence catchment area**								
Centro #	1.00				1.00			
Guadalmedina	-0.07	0.12	-0.56	0.57	0.05	0.08	0.64	0.52
Outside study area	-0.32	0.17	-1.87	0.06	-0.17	0.10	-1.80	0.07
**Residence municipality by rurality factorÇ**								
Malaga (city) (-1.78: less rurality) #	1.00				1.00			
Rincón de la Victoria (-1.78)	-0.19	0.18	-1.04	0.30	-0.18	10.00	-1.68	0.09
Other villages (-1.59 to - 0.61: more rurality)	-0.38	0.18	-2.03	0.04**	-0.24	0.13	-1.82	0.07
Outside area	-0.31	0.15	-2.09	0.04**	Omitted&			
**Clinical diagnoses (ICD-10)**								
F20 Schizophrenia #	1.00				1.00			
F22 Persistent delusional disorders	-0.51	0.08	-6.00	0.00**	-0.15	0.07	-2.03	0.04**
F23 Acute and transient psychotic disorders	-0.07	0.11	-0.61	0.54	0.10	0.09	1.11	0.27
F25 Schizoaffective disorders	0.20	0.12	1.68	0.09	0.06	0.09	0.62	0.54
F21,F24,F28 Y F29 @	-0.28	0.15	-1.84	0.06	-0.06	0.12	-0.54	0.59
**Global Level of Severity**								
Level I (less severity) #	1.00				1.00			
Level II	0.40	0.70	5.64	0.00**	0.23	0.61	3.78	0.00**
Level III	0.97	0.11	8.87	0.00**	0.45	0.94	4.79	0.00**
Level IV (more severity)	0.74	0.21	3.48	0.00**	0.35	0.18	1.92	0.06
**Type of professional who attended patients**								
Only psychiatrists #	1.00				1.00			
Only nurses	0.22	0.10	2.18	0.03**	0.02	0.10	0.24	0.81
Both (psychiatrists and nurses)	1.31	0.53	24.98	0.00**	1.09	0.06	19.56	0.00**
Other professionals	-0.78	0.29	-2.65	0.01**	-0.91	0.29	-3.10	0.00**
**Psychiatrist who attended patients**								
C #	1.00				1.00			
B	0.00	0.13	0.00	0.99	-0.08	0.10	-0.76	0.45
L	-0.29	0.20	-1.47	0.14	-0.30	0.16	-1.90	0.06
H	0.26	0.14	1.81	0.07	0.13	0.11	1.14	0.26
E	-0.42	0.14	-3.00	0.00**	-0.50	0.11	-4.49	0.00**
D	-0.15	0.15	-0.99	0.32	-0.20	0.12	-1.71	0.09
K	-0.36	0.20	-1.79	0.07	-0.25	0.16	-1.61	0.11
F	-0.22	0.15	-1.42	0.16	-0.35	0.12	-2.83	0.00**
G	-0.28	0.15	-1.81	0.07	-0.19	0.13	-1.49	0.14
I	-0.36	0.20	-1.78	0.08	-0.40	0.14	-2.78	0.00**
A	-0.37	0.14	-2.63	0.01**	-0.26	0.11	-2.32	0.02**
J	-0.36	0.19	-1.92	0.06	-0.16	0.16	-1.00	0.32
M	-0.17	0.23	-0.75	0.45	0.08	0.18	0.41	0.68
**Number of inpatient psychiatric admissions**								
0 admissions #	1.00				1.00			
1 admission	1.00	0.16	6.36	0.00**	1.18	0.36	3.25	0.00**
2+ admissions	1.26	0.20	6.43	0.00**	1.12	0.29	3.88	0.00**
**Number of readmissions**								
0 admissions, 0 readmissions #	1.00				1.00			
Any inpatient admission, 0 readmissions	1.04	0.14	7.53	0.00**	-0.45	0.30	-1.53	0.12
Any inpatient admission, 1+ readmissions	1.33	0.27	4.92	0.00**	Omitted&			
**Number of days in hospital**								
0 day #	1.00				1.00			
<19 days	1.07	0.15	7.06	0.00**	-0.26	0.21	-1.25	0.21
19+ days	1.16	0.21	5.55	0.00**	Omitted&			

Multilevel linear regression with imputed data (20 data sets). Adjusted coefficients of the full model (including all variables)

# Reference groups in all references; Ç rurality index developed for the Spanish population which has range of -3.59 (minimum) to 3.78 (maximum);

@ F21 = Schizotypal disorder, F24 = Induced delusional disorder, F28 = other non-organic psychotic disorders and F29 = Unspecified non-organic psychosis;

*&*Omitted because of collinearity. Coef: Coeficient Beta; SE: Standard Error; t: experimental value; Transformed by logarithm (x+1). Figures in bold (**) P < 0.05.

## Discussion

The average age of our study sample was 44 years and the Global Severity Index was one (out of four), i.e., patients who were clinically stable, without residual symptoms, with good social and family adjustment, autonomous or with minimal dependency and in regular contact with services. The average number of outpatient contacts per year was 11.14. Despite this global severity index, only 18% were working. In Spain, as in other European countries, this employment status is common in persons with schizophrenia, with rates ranging between 10% and 20% [30,31].

This study used a linear regression approach to examine different factors (such as the socio-demographic and clinical characteristics of the patients and their use of services) possibly related with the use of community mental health services. This multilevel analysis in which adjustment was made for possible effects of clustering explained nearly 50% (46.35%) of the variance in contacts with the outpatient mental health services. In the field of public health this approach is considered a very good explicative model.

After controlling for socio-demographic, clinical, professional and service use variables, no gender differences were found concerning the use of outpatient services, similar to the results of Lindamer *et al. *[10]. Unlike some reports [8,9], our findings suggest that age is not associated with the number of outpatient contacts, though this may be related to under-representation of elderly patients. Variables concerning type or place of residence showed slight significance (p < 0.05).

After adjustment, the number of outpatient contacts was positively associated with no formal education and not working, receiving welfare benefits. It is well known that patients receiving welfare benefits are heavy users of outpatient mental health services [8]. The first reason for this is that the illness of these persons is more severe than those who are working. Another reason could be that welfare benefits need to be revised routinely according to information supplied by the psychiatrist. Yet another reason concerns the fact that persons who do not work have more free time to attend health services. The level of education can also play a role, and our results show that a lower educational attainment increases the risk of higher service use. This could be due to less tolerance or knowledge of the illness and to an increased need to seek help. Nevertheless, our results concerning level of education differ from those found by Pezzimenti *et al. *[4] and Cooper-Patrick *et al. *[32], where a high level of education was associated with greater use of ambulatory services.

As expected, an increase in the level of severity was generally associated with a greater frequency of outpatient contacts [8,33]. This group of patients presents frequent decompensation, moderate disability and predominantly negative symptoms. But what is important to highlight, concerning the efficiency of the attention, is that the group with the highest severity level (level IV: higher disability, without support and with serious symptoms), showed a weaker association with frequency of use (p = 0.06), probably due to lower adherence to treatment in this group. The strong relationship between having inpatient admissions and a higher number of outpatient contacts implies that patients who had been in hospital were receiving continuity of care because of decompensation of the illness, reflected in a higher number of contacts with community services. This result is similar to the findings by Kent *et al. *[34] and Roick *et al. *[35], which showed that frequent users of psychiatric inpatient care also consume more outpatient services. The particular psychiatrist attending a patient was strongly associated with the total number of outpatient contacts. As the type of patient was similar for all the psychiatrists, this result could be a consequence of differences in the characteristics of each professional related, for example, to his or her length of clinical experience, professional orientation, the case load size or burnout. Some studies have suggested that psychiatrists have higher levels of burnout than other physicians employed in general medical settings [36-38]. This finding, called "induced demand by the professional", is well known in the field of health services and in health economics [39], but as far as we know, no study in the psychiatric area has focused on patients with schizophrenia and analyzed the effect of the attending psychiatrist as a possible factor related with differences in outpatient contacts. Finally, the strongest association was with the type of professional seeing the patient. The patients who had contacts with both types of professional, nurses and psychiatrists, had significantly more contacts with the community centre than the patients who only had contacts with psychiatrists. A possible explanation for this concerns the profile of patients receiving combined treatment (both psychiatric and nursing), who usually have a low functioning level and more problems in basic everyday living skills, with more chronic and negative symptoms [40]. Nurses play a relevant role in the care of patients with schizophrenia, centred mainly on achieving maximum patient autonomy and adaptation to the social environment through control of antipsychotic medication or with psychosocial interventions [41]. In addition, many nurses visited their clients at their place of residence. Some studies have shown that the structure of the service delivery system [42] and staff can be major determinants of heavy psychiatric service use [18], though this has mainly been studied in hospital settings [34]. Lemming and Calsyn [43] showed that social support from professionals was the strongest predictor for service utilization in persons with severe mental illness.

### Strengths and limitations of the study

This study has several strong points. The analyses are based on a large sample of data extracted from a Mental Health Information System (RESMA). The homogeneity of the study population (users with schizophrenia or related disorders living in the community) provides a non-biased picture of the overall use of clinical services, and not just for patients with previous hospitalization, as seen in most studies. Another strong point is that the study includes an operationalized definition of the use of services, defined as the number of contacts with outpatient mental health services, which we calculated after excluding the number of days each patient had been hospitalized in a psychiatric ward. This provides more realistic information about use of services. Furthermore, we used multilevel linear regression with two levels, patients and primary care centres.

However, before accepting the validity of our results, it is important to consider some limitations. First, and perhaps most importantly, this was a cross-sectional study. Accordingly, we cannot infer causality but only association between factors and our dependent variable. Secondly, the diagnoses were clinical (no structured interviews were used) and the validity of the Global Level of Severity is unknown. However, these assessments were done by specialized psychiatrists who care for patients over a long time [10] and for the GLS we calculated test-retest agreement at two times, with a good coefficient according to Streiner [44]. Third, as the sources of data in this study were routine clinical databases, reliability of data completion in the clinical practice cannot be assured given the difficulties this task sometimes involves in this setting. However, concerning this matter, we used an imputation method to compensate for the missing data. Finally, as data were recorded in the public service setting, care provided by private psychiatrists or psychologists was not included. Nevertheless, in psychotic patients, a progressive shift of patients from the private sector to the public systems occurs as the illness becomes chronic [45]. Thus, we can assume that the sample was representative of the patients with schizophrenia and related disorders in Malaga, always considering a similar Global Level of Severity.

## Conclusions

Our model explained a larger percentage of the estimated variance than most studies on the use of community mental health services by patients with schizophrenia or related disorders. As expected, the variables that explained the use of community service could be viewed as being proxies for severity of illness.

The most surprising finding of this study was that the psychiatrists who care for schizophrenic patients in organized outpatient mental health settings are themselves associated with the number of contacts with these ambulatory services. The complexity that characterizes treatment of schizophrenic disorders in the community setting shows that further research should focus on mental health professionals, who now seem to be a relevant factor not previously considered in international studies of service use by patients with schizophrenia. More research is needed to carefully examine how professional support networks interact to affect use of mental health.

### Ethical approval

Approval for the study was obtained from the Carlos Haya Hospital Ethics Committee.

## Competing interests

The authors declare that they have no competing interests.

## Authors' contributions

BMK is guarantor for the study. BMK, FM and IG designed the study and the other authors collaborated in the design. BMK, JAB, PMP and OP analyzed the data and the other authors contributed to the approach to the analysis. BMK coordinated the study and FR, PA, JR, JMGH, FM and DN collaborated implementing the study in each mental health service. BMK and AS drafted the paper and all authors discussed and agreed the final version.

## Pre-publication history

The pre-publication history for this paper can be accessed here:

http://www.biomedcentral.com/1472-6963/11/257/prepub

## References

[B1] Gater R Amaddeo F Tansella M Jackson G Goldberg D A Comparison of Community-Based Care for Schizophrenia in South-Verona and South Manchester Br J Psychiatry 1995 166 344 352 10.1192/bjp.166.3.344 7788126

[B2] Haro JM Salvador-Carulla L Cabasés J Madoz V Vazquez-Barquero JL Utilisation of Mental Health Services and Costs of Patients with Schizophrenia in three areas of Spain Br J Psychiatry 1998 173 334 340 10.1192/bjp.173.4.334 9926039

[B3] Lora A Cosentino U Gandini A Zocchetti C Which Community Care for Patients with Schizophrenic Disorders?. Packages of Care Provided by Departments of Mental Health in Lombardy (Italy) Epidemiologie e Psichiatria Sociale 2007 16 300 338 18333429

[B4] Pezzimenti M Haro JM Ochoa S González JL Almenara J Alonso J Moreno B Muñoz PE Jáuregui VM Salvador-Carulla L the PSICOST Group Assessment of Service Use Patterns in Out-Patients with Schizophrenia, a Spanish Study Acta Psychiatr Scand 2006 114 Suppl 432 12 8 10.1111/j.1600-0447.2006.00915.x 17087811

[B5] Salvador-Carulla S Tibaldi G Hohnson S Scala E Romero C Munizza C Patterns of Mental Health Service Utilisation in Italy and Spain Soc Psychiatry Psychiatr Epidemiol 2005 40 149 159 10.1007/s00127-005-0860-y 15685407

[B6] Tansella M Amaddeo F Burti L Lasalvia A Ruggeri M Evaluating a Community-Based Mental Health Service Focusing on Severe Mental Health Illness. The Verona Experience Acta Psychiatr Scand 2006 113 Suppl 429 90 94 10.1111/j.1600-0447.2005.00724.x 16445489

[B7] Becker T Vázquez-Barquero JL The European Perspective of Psychiatry Reform Acta Psychiatr Scand 2001 104 Suppl 410 8 14 10.1034/j.1600-0447.2001.1040s2008.x 11863056

[B8] Carr V Johnston PJ Lewin T Rajkumar S Carter GL Issakidis C Patterns of Service use Among Persons with Schizophrenia and Other Psychotic Disorders Psychiatr Serv 2003 54 226 235 10.1176/appi.ps.54.2.226 12556605

[B9] Jin H Folsom D Lindamer L Bailey A Hawthorne W Garcia P Jeste DV Patterns of Public Mental Health Service Use by Age in Patients with Schizophrenia Am J Geriatr Psychiatry 2003 11 525 533 14506086

[B10] Lindamer LA Bailey A Hawthorne W Folsom DP Gilmer TP Garcia P Hough RL Jeste DV Gender Differences in Characteristic and Service Use of Public Mental Health Patients with Schizophrenia Psychiatr Serv 2003 54 1407 1409 10.1176/appi.ps.54.10.1407 14557530

[B11] Barrio C Yamada AM Hought Rl Hawthorne W Garcia P Jeste DV Ethnic Disparities in Use of Public Mental Health Management Services Among Patients with Schizophrenia Psychiatr Serv 2003 54 1264 1270 10.1176/appi.ps.54.9.1264 12954944

[B12] Folsom D Gilmer T Barrio C Moore DJ Bucardo J Lindamer LA Garcia P Hawthorne W Hough R Patterson T Jeste DV A Longitudinal Study of the Use of Mental Health Services by Personas with Serious Mental Illness: Do Spanish-Speaking Latinos Differ from English-Speaking Latinos and Caucasians? Am J Psychiatry 2007 164 1173 1180 10.1176/appi.ajp.2007.06071239 17671279

[B13] Tello JE Mazzi M Tansella M Bonizzato P Jones J Amaddeo F Does Socieconomic Status Affect the Use of Community-Based Psychiatric Services? A South Verona Case Register Study Acta Psychiatr Scand 2005 112 215 223 10.1111/j.1600-0447.2005.00558.x 16095477

[B14] Moreno B Arroyo B Torres-González F Luna JD Cervilla J Social Predictors of Out-Patient Mental Health Contact in Schizophrenia Patients Soc Psychiatry Psychiatr Epidemiol 2007 42 452 456 10.1007/s00127-007-0187-y 17473903

[B15] Drukker M Driessen G Krabbendam L Os JV The Wider Social Environment and Mental Health Service Use Acta Psychiatr Scand 2004 110 119 129 10.1111/j.1600-0047.2004.00341.x 15233712

[B16] McCarthy JF Piette JD Fortney JC John C Valenstein M Blow F Outpatient Visit Chaining Among Patients with Serious Mental Illness Med Care 2006 44 257 264 10.1097/01.mlr.0000199661.94141.b8 16501397

[B17] McCreadie RG Leese M Tilak-Singh D Loftus L MacEwan T Thornicroft G Nithsdale, Nunhead and Norwood: Similarites and Differences in Prevalence of Schizophrenia and Utilisation of Services in Rural and Urban Areas Br J Psychiatry 1997 170 31 36 10.1192/bjp.170.1.31 9068772

[B18] Fleury MJ Grenier G Bambita JM Caron J Professional Service Utilisation among Patients with Severe Mental Disorders BMC Health Serv Res 2010 10 141 20507597 10.1186/1472-6963-10-141 PMC2896947

[B19] Becker T Kilian R Psychiatric Services for People with Severe Mental Illness Across Western Europe: What can be Generalized from Current Knowledge about Differences in Provision, Costs and Outcomes of Mental Health Care? Acta Psychiatr Scand 2006 113 Suppl 429 9 16 10.1111/j.1600-0447.2005.00711.x 16445476

[B20] Monzani E Erlicher A Lora A Lovaglio P Vittadini G Does Community Care Work? A Model to Evaluate the Effectiveness of Mental Health Services Int J Mental Health Systems 2008 2 10 10.1186/1752-4458-2-10 PMC2488329 18601741

[B21] Tzeng DS Lian LC Chang CU Yang CY Lee GT Pan P Lung FW Health in schizophrenia: effectiveness and progress of a redesigned care network BMC Health Serv Res 2007 7 129 10.1186/1472-6963-7-129 17705853 PMC2000889

[B22] Moreno B Mayoral F Pérez O García-Herrera JM Algarra J Rivas F Pérez R Becerra F Gornemann I The Málaga Schizophrenia Case-Register (Resma): Overview of Methodology and Patient Cohort Int J Soc Psychiatry 2009 55 5 15 10.1177/0020764008092237 19129322

[B23] Ocaña-Riola R Sánchez-Cantalejo E Rurality Index For Small Areas In Spain Soc Indic Res 2005 73 247 266 10.1007/s11205-004-0987-3

[B24] Prieto-Lara E Ocaña-Riola R Updating Rurality Index For Small Areas In Spain Soc Indic Res 2010 95 267 280 10.1007/s11205-009-9459-0

[B25] World Health Organization The ICD-10 Classification of Mental and Behavioural Disorders: Diagnostic criteria for research 1993 Geneva, World Health Organization

[B26] Valmisa Gómez De Lara E II Plan Integral De Salud Mental 2008-2012 2008 Sevilla: Consejería de Salud de la Junta de Andalucía

[B27] Statacorp LP Stata: Release 11. Statistical Software 2009 Texas: College Station

[B28] Kenward MG Carpenter JR Multiple Imputation: Current Perspectives Stat Methods Med Res 2007 16 199 218 10.1177/0962280206075304 17621468

[B29] Little RJA Rubin DB Statistical Analysis with Missing Data 2002 2 New York: Wiley

[B30] Gaite L Vázquez-Barquero JL Borra C Ballesteros J Schene A Welcher B Thornicroft G Becker T Ruggeri M Herran A Quality of life in patients with schizophrenia in five Europena countries: the EPSILON study Acta Psychiatr Scand 2002 105 283 292 10.1034/j.1600-0447.2002.1169.x 11942933

[B31] Marwaha S Johnson S Schizophrenia and employment Soc Psychiatry Psychiatr Epidemiol 2004 39 337 349 10.1007/s00127-004-0762-4 15133589

[B32] Cooper-Patrick L Gallo JJ Powe NR Steinwachs DM Eaton WW Ford DE Mental health service utilization by African Americans and Whites Med Care 1999 10 1034 1045 10.1097/00005650-199910000-00007 10524370

[B33] Lora A Cosentino U Rossin MS Lanzara D A Cluster Analysis of Patients with Schizophrenia in Community Care Psychiatr Serv 2001 52 682 684 10.1176/appi.ps.52.5.682 11331807

[B34] Kent S Fogarty M Yellowlees P A Review of Studies of Heavy Users of Psychiatric Services Psychiatr Serv 1995 46 1247 1253 8590109 10.1176/ps.46.12.1247

[B35] Roick C Heider D Kilian R Matschinger H Toumi M Anermeyer MC Factors contributing to frequent use of psychiatric inpatient services by schizophrenia patients Soc Psychiatry Psychiatr Epidemiol 2004 39 744 751 10.1007/s00127-004-0807-8 15672296

[B36] Bressi C Porcellana M Gambini O Madia L Muffatti R Peirone A Zanini S Erlicher A Scarone S Altamura AC Burnout among psychiatrists in Milan: a multicenter survey Psychiatr Serv 2009 60 985 8 10.1176/appi.ps.60.7.985 19564233

[B37] Kumar S Hatcher S Dutu G Fischer J Ma'u E Stresses experienced by psychiatrists and their role in burnout: a national follow-up study Int J Soc Psychiatry 2011 57 166 179 10.1177/0020764009341211 20068020

[B38] Kumar S Burnout in psychiatrists World Psychiatry 2007 6 186 189 18188444 PMC2175073

[B39] Bellón JA Martinez-Cañavate T Delgado A Luna JD Modelo Multinivel Explicativo de la Utilización de las Consultas de Atención Primaria a Partir de los Factores del Profesional y la Organización Atención Primaria 2004 34 137 811844426

[B40] Quilez J Foix A Ramon J Monsterrat M Farreras P Montes C Santos M Miguel J NEDES group Pacientes esquizofrénicos que viven en la comunidad. Derivación a enfermería Rol de Enfermería 2003 26 278 282 14502933

[B41] Gournay K Role of the community psychiatric nurse in the management of schizophrenia Adv Psychiatr Treat 2000 6 243 249 10.1192/apt.6.4.243

[B42] Alvarez B La Demanda Atendida de Consultas Médicas y Servicios Urgentes en España Investigaciones Económicas 2001 XXV 93 138

[B43] Lemming MR Calsyn RR Utility of the behavioral model in predicting service utilization by individuals suffering from severe mental illness and homelessness Community Ment Hlt J 2004 40 347 364 10.1023/b:comh.0000035229.20557.5c 15453086

[B44] Streiner Dl Norman GR Health Measurement Scales 1989 Oxford: Oxford University Press

[B45] Baca Baldomero E Leal Cerc ós C Valera C Riesgo Y Roca M Diagnóstico y Manejo de la Esquizofrenia en España: El Proyecto ACEE Actas Esp Psiquiatri 2006 34 224 230 16823682

